# Reduction of Hospital-Acquired Infections Through a Nursing Education Program: A Quality Improvement Project on the Sensitization of Nursing Staff Toward Infection Control in Neonates

**DOI:** 10.7759/cureus.62656

**Published:** 2024-06-18

**Authors:** DurreShahwar Khan, Fatima Waqar, Nazish Azim, Owais Khan, Amir Sohail

**Affiliations:** 1 Neonatology, Liaquat National Hospital and Medical College, Karachi, PAK; 2 Pediatrics, Liaquat National Hospital and Medical College, Karachi, PAK; 3 Pediatrics and Child Health, Liaquat National Hospital and Medical College, Karachi, PAK; 4 Internal Medicine, Liaquat National Hospital and Medical College, Karachi, PAK

**Keywords:** bloodstream infection, neonatal intensive care unit (nicu), neonates, quality improvement project, hospital-acquired infections

## Abstract

Background

This study aimed to reduce hospital-acquired infections (HAIs) by at least 50% in our unit through a nursing education program to sensitize the nursing staff toward infection control in neonates.

Methodology

This pre- and post-intervention observational study was conducted in Liaquat National Hospital’s Neonatal Intensive Care Unit (NICU) from October 2021 until March 2023. This observational study was conducted in three phases. In phase I (pre-implementation), all neonates with suspected HAIs were included. In phase II (implementation), the nursing staff dedicated to the NICU were trained. In phase III (post-implementation), all neonates with suspected HAIs were included. Culture-proven bloodstream infections (BSIs), urinary tract infections (UTIs), and ventilator-associated pneumonia (VAP) were the three types of HAIs. The nursing scoring was done by the principal investigator based on a questionnaire. The Shapiro-Wilk test was used to evaluate the normality of all quantitative data across all phases.

Results

In the pre-implementation phase, there were 24 (10.8%) HAIs, among which 19 (8.6%) were BSIs, one (0.5%) was a catheter-associated urinary tract infection (CAUTI), and seven (3.2%) were VAP. Whereas in the post-implementation phase, there were 12 (5%) HAIs, among which 10 (4.1%) were BSIs, none were CAUTIs, and five (2.1%) were VAP. There was a significant reduction of HAIs in the post-implementation phase (p < 0.01). The difference in the knowledge, assessment, and practice was statistically significant in the post-implementation phase (p < 0.01).

Conclusions

We established a successful cost-effective intervention to improve the awareness and compliance of NICU nurses with infection control practices. This helped us in reducing HAIs in our NICU.

## Introduction

Hospital-acquired infections (HAIs) remain a concern of utmost importance in neonatal intensive care units (NICUs) [1]. HAIs contribute to a large number of neonatal morbidity and mortality [1,2]. The most common causes of infections include a lack of standard hand hygiene practices and central line-associated bloodstream infections (CLABSIs) [1,2]. Other HAIs include catheter-induced urinary tract infections (CAUTIs) and ventilator-associated pneumonia (VAP).

According to a study, an education program particularly for intensive care unit (ICU) nurses significantly lowered the frequency of primary bloodstream infections (BSIs) [3]. Another study suggested a significant decline in the incidence of VAP by nursing education [4,5]. Literature suggests a strong association of HAIs with hand hygiene [6,7]. A multicenter meta-analysis suggested the importance of environmental cleaning in preventing HAIs [8].

According to the World Health Organization, bacterial infections are responsible for 25% of newborn deaths each year [9]. HAIs, with prevalence ratios in lower-middle-income countries (LMICs) being 3 to 20 times higher, are a substantial contributor to neonatal morbidity and mortality [10]. One of the Sustainable Development Goals aims to lower newborn mortality to below 12 per 1,000 live births globally by 2030 [11]. Due to population expansion, in-hospital births, and preterm delivery rates in Southeast Asia, neonatal HAI has a significant burden and is predicted to worsen [12].

Given the above evidence and increasing neonatal mortality secondary to infection, we initiated a regular nursing education program dedicated to the NICU nursing staff. The topics discussed were common practices in NICU such as hand hygiene, suction (endotracheal tube/oral/nasal) practices, line handling, skincare, temperature regulation, intravascular access, phlebitis prevention and management, blood sampling techniques, preterm care, neonatal hypothermia and its prevention, and incubator care. The predictors of outcome were the rate of infection in the pre-implementation phase and post-implementation phase. The aim was to improve the healthcare delivery to the patients admitted to the NICU of our center.

## Materials and methods

This pre- and post-intervention observational study was conducted at Liaquat National Hospital’s NICU from October 2021 to March 2023. This study was approved by the Ethical Review Committee, Liaquat National Hospital and Medical College (reference number: LNH-Pds-Med-02/2023/14). A quality improvement (QI) team was established for nursing neonatal care training, including neonatologists, an infectious disease consultant, and a pediatrician. Every educational lecture was for one hour and was given by the consultants.

Inclusion and exclusion criteria

All 27 nursing staff designated in the NICU were included in the study. In the pre-implementation phase, we included all neonates who were suspected to have HAIs, with a sample size of 225. Similarly, in the post-implementation phase, we included all neonates having suspicion of HAIs, with a sample size of 242. All neonates with early-onset sepsis or whose initial (sent on admission) blood culture was positive were excluded from the study.

The study was conducted in three phases. In phase I (pre-implementation), all patients who were suspected of HAIs were included. To retrieve data the hospital’s electronic medical record system was employed. In phase II (implementation), the training was conducted. In every group, eight nurses were enrolled. The attendance of the participants was kept mandatory. Each session was led personally by the QI team. For nursing scoring, the primary investigator (PI) distributed the same questionnaire (as of the pre-implementation phase) to the same nurses. In phase III (post-implementation), all patients who were suspected of having HAIs were included.

A structured questionnaire was designed for nursing scoring in the pre-implementation and post-implementation phases. The questionnaire consisted of 10 questions. A total of five questions were on knowledge, three on attitude, and two on practices. Correct and erroneous answers to knowledge questions were marked with a 1 or 0, while attitudes and practices were graded on a Likert scale ranging from 1 to 5. A score of 7-10 was considered good, 5-7 was considered fair, and <5 was considered poor.

HAIs were categorized as culture-proven BSI, UTI, and VAP. We recorded the date of admission and the date of cultures that became positive after 48 hours of admission in the NICU, after 48 hours of bladder catheterization, and/or after 48 hours of ventilatory support. We also collected data for patient days, outcomes such as survival or death, and organisms (bacteria, viruses, or fungi) reported. The nursing scoring was done by the PI based on a questionnaire. For data collection and analysis throughout all phases, SPSS Statistics version 23 (IBM Corp., Armonk, NY, USA) was utilized. In all phases, the normality of all quantitative variables was determined using the Shapiro-Wilk test. The descriptive statistics were calculated.

Pre-implementation and post-implementation phase statistics

Overall mean, standard deviation, or median and interquartile range (IQR) (if data do not follow normality) were computed for quantitative variables, i.e., gestational age, birth weight, length, front occipital circumference (FOC), total patient days, and nursing score. Frequency and percentage were calculated for qualitative variables, i.e., BSI, CAUTI, VAP, bacteria, virus, fungus, organism, outcome, and HAI. Stratification was done for gestational age, birth weight, length, FOC, HAI, BSI, CAUTI, VAP, bacteria, virus, fungus, total patient days and knowledge, attitude, and practice level by the nursing score to see the effect of these modifiers on HAIs. To determine the relationship between HAI and qualitative characteristics, the chi-square test and Fisher’s exact test were utilized. Independent-sample t-test or Mann-Whitney U test (as appropriate) were used to see the significant difference of mean in qualitative variables according to HAIs. P-values ≤0.05 were considered significant in all analyses

Further, the pre- and post-implementation phases were compared. The association of outcome, i.e., HAI among both phases was evaluated using McNamara’s test. The significance of HAIs among both phases according to quantitative and quantitative variables was evaluated using the chi-square test/Fisher’s exact test, independent-sample t-test or Mann-Whitney U test, paired-sample t-test, or Wilcoxon signed-rank test.

SPSS was used to store and analyze the data. Mean with standard deviation were reported for gestational age, birth weight, length, FOC, and total patient days. Counts with percentages were reported for HAI, BSI, CAUTI, VAP, bacteria, viruses, fungi, and questions on knowledge, attitude, and practice (KAP) assessment. Independent-sample t-test was used to compare the means between pre- and post-implementation groups. The chi-square test was used to check the association of HAI, BSI, CAUTI, VAP, bacteria, virus, and fungi outcomes in pre- and post-implementation groups. The Mann-Whitney U test was used to compare the outcomes between pre- and post-implementation groups. Wilcoxon signed-rank test was used to compare KAP between samples within the pre- and post-implementation phases. The results of the study were graphically shown using bar graphs, and p-values less than 0.05 were considered statistically significant. Median with IQR was reported for gestational age, birth weight, length, FOC, and total patient days. Kaplan-Meier test was used to report the survival curve and estimate the survival period of samples. Survival curves of pre- and post-implementation were compared using the log-rank test.

## Results

A total of 463 neonates were evaluated. There were 222 neonates in the pre-implementation phase and 241 neonates in the post-implementation phase.

In the pre-implementation phase, the median gestational age (weeks) was 35 (IQR = 37-33], the median birth weight (kg) was 2.3 (IQR = 2.78-1.8), the median length (cm) was 49 (IQR = 50-46), the median FOC (cm) was 33 (IQR = 33-32), and the median total patient days were 3 (IQR = 7-2). In the post-implementation phase, the median gestational age (weeks) was 36 (IQR = 37-34), the median birth weight (kg) was 2.46 (IQR = 2.82-1.96), the median length (cm) was 46 (IQR = 47-44), the median FOC (cm) was 34 (IQR = 35-32), and the median total patient days were 3 (IQR = 6-3). There was a significant difference in the median of gestation age, length, and FOC at pre- and post-implementation (p < 0.05).

As shown in Table 1, in the pre-implementation phase, 24 (10.8%) had HAIs, 19 (8.6%) had BSIs, one (0.5%) had CAUTI, and seven (3.2%) had VAP. Among organisms, 21 (9.5%) were bacteria, and eight (3.6%) were fungi. In the post-implementation phase, 12 (5%) had HAIs, 10 (4.1%) had BSIs, none (0%) had CAUTI, and five (2.1%) had VAP. Among organisms, 11 (4.6%) were bacteria, and one (0.4%) was fungus. There was a significant decrease in HAIs, bacteria, and fungi in the post-implementation phase (p < 0.05).

**Table 1:** HAI, BSI, CAUTI, VAP, bacteria, and fungi in the pre- and post-implementation phases (n = 463). *: P < 0.05 was considered statistically significant using the Mann-Whitney test. HAI = hospital-acquired infection; BSI = bloodstream infection; CAUTI = catheter-associated urinary tract infection; VAP = ventilator-associated pneumonia

Parameters		Group				P-value
		Pre-implementation		Post-implementation		
		n	%	n	%	
HAI	Yes	24	10.8	12	5.0	0.01*
	No	198	89.2	229	95.0	
BSI	Yes	19	8.6	10	4.1	0.051
	No	203	91.4	231	95.9	
CAUTI	Yes	1	0.5	0	0.0	0.29
	No	221	99.5	241	100.0	
VAP	Yes	7	3.2	5	2.1	0.46
	No	215	96.8	236	97.9	
Bacteria	Yes	21	9.5	11	4.6	0.03*
	No	201	90.5	230	95.4	
Fungi	Yes	8	3.6	1	0.4	0.01*
	No	214	96.4	240	99.6	

Regarding the comparison of the outcomes, in the pre-implementation phase, 204 (91.9%) patients survived, and 18 (8.1%) expired. In the post-implementation phase, 223 (92.5%) survived and 18 (7.5%) expired. Overall, the results showed improved survival in the post-implementation phase; however, it was not statistically significant (p = 0.79).

The mean survival days in the pre-implementation phase were 7.3 (SE = ±0.7), and in the post-implementation phase, it was 7.5 (SE = ±0.8); however, no significant difference was observed for the survival curves of pre- and post-implementation samples (p = 0.63).

Table 2 presents the comparison of knowledge with pre- and post-implementation samples. In the pre-implementation phase, 21 (87.5%) agreed that preterm babies are more susceptible to infections than term babies. Overall, 21 (87.5%) agreed that hypothermia increases mortality and morbidity in neonates. A total of 22 (91.7%) participants agreed that intravenous (IV) cannulation should be done under sterile techniques. About 22 (91.7%) participants agreed that umbilical catheterization not done under a strict sterile technique can lead to CLABSIs. About six (25%) participants agreed that for blood sampling in a newborn, the puncture site should be warm and sterile.

**Table 2:** Comparison of knowledge with pre- and post-implementation samples. *: P < 0.05 was considered statistically significant using the Wilcoxon signed-rank test. CLABSI = central line-associated bloodstream infection

Knowledge		Pre-implementation (n = 24)		Post-implementation (n = 24)		P-value
		n	%	n	%	
Preterm babies are more susceptible to infections than term babies	Yes	21	87.5	23	95.8	0.31
	No	3	12.5	1	4.2	
Hypothermia increases mortality and morbidity in neonates	Yes	21	87.5	22	91.7	0.65
	No	3	12.5	2	8.3	
Intravenous cannulation should occur under a sterile technique	Yes	22	91.7	21	87.5	0.56
	No	2	8.3	3	12.5	
Umbilical catheterization not occurring under a strict sterile technique can lead to CLABSI	Yes	22	91.7	19	79.2	0.18
	No	2	8.3	5	20.8	
For blood sampling in a newborn, the puncture site should be warm and sterile	Yes	6	25.0	20	83.3	<0.01*
	No	18	75.0	4	16.7	

Whereas in the post-implementation phase, 23 (95.8%) agreed that preterm babies are more susceptible to infections than term babies. A total of 22 (91.7%) participants agreed that hypothermia increases mortality and morbidity in neonates. Overall, 21 (87.5%) participants agreed that IV cannulations should occur under a sterile technique. About 19 (79.2%) participants agreed that umbilical catheterization not done under a strict sterile technique can lead to CLABSIs, and 20 (83.3%) agreed that for blood sampling in a newborn, the puncture site shall be warm and sterile.

There was a significant difference in the knowledge that for blood sampling in a newborn, the puncture site should be warm and sterile between the pre- and post-implementation samples (p < 0.01).

Table 3 presents the comparison of attitude in the pre-and post-implementation samples. Overall, 29.2% said often hand hygiene measures the risk of infection among patients. Regarding a non-sterile technique of airway suction that can lead to HAIs among patients, 37.5% said sometimes. Further, 66.7% said always to cleaning patients’ belongings is mandatory to prevent HAIs. In the post-implementation phase, 58.3% said always to hand hygiene measures the risk of infection among patients. Moreover, 83.3% said always to the non-sterile technique of airway suction can lead to HAIs among patients, and 79.2% said always to cleaning patients’ belongings is mandatory to prevent HAIs. There was a significant difference in attitude during the post-implementation phase for hand hygiene and non-sterile technique (p < 0.05).

**Table 3:** Comparison of attitudes with pre- and post-implementation samples. *: P < 0.05 was considered statistically significant using the Wilcoxon signed-rank test.

Attitude		Pre-implementation (n = 24)		Post-implementation (n = 24)		P-value
		n	%	n	%	
Hand hygiene measures the risk of infection among patients	Always	4	16.7	14	58.3	<0.01*
	Often	7	29.2	7	29.2	
	Sometimes	0	0.0	1	4.2	
	Rarely	8	33.3	1	4.2	
	Never	5	20.8	1	4.2	
The non-sterile technique of airway suction can lead to hospital-acquired infections among patients	Always	2	8.3	20	83.3	<0.01*
	Often	0	0.0	2	8.3	
	Sometimes	9	37.5	0	0.0	
	Rarely	7	29.2	0	0.0	
	Never	6	25.0	2	8.3	
Cleaning of patients’ belongings is mandatory to prevent hospital-acquired infections	Always	16	66.7	19	79.2	0.06
	Often	1	4.2	3	12.5	
	Sometimes	0	0.0	0	0.0	
	Rarely	3	12.5	0	0.0	
	Never	4	16.7	2	8.3	

Table 4 presents the comparison of practice with pre- and post-implementation samples. In the pre-implementation phase, 33.3% said they often perform a physical examination of the cannulation site before giving injections, and 70.8% said they always clean the incubator of their patient daily.

**Table 4:** Comparison of practice with pre- and post-implementation samples. *: P < 0.05 was considered statistically significant using the Wilcoxon signed-rank test.

Practice		Pre-implementation (n = 24)		Post-implementation (n = 24)		P-value
		n	%	n	%	
I do a physical examination of the cannulation site before giving injections	Always	3	12.5	16	66.7	0.02*
	Often	8	33.3	5	20.8	
	Sometimes	10	41.7	0	0.0	
	Rarely	2	8.3	0	0.0	
	Never	1	4.2	3	12.5	
I clean the incubator of my patient daily	Always	17	70.8	21	87.5	0.06
	Often	0	0.0	1	4.2	
	Sometimes	0	0.0	0	0.0	
	Rarely	5	20.8	0	0.0	
	Never	2	8.3	2	8.3	

In the post-implementation phase, 66.7% said they always perform a physical examination of the cannulation site before giving injections, and 87.5% said they always clean the incubator of their patient daily. The difference in practice on physical examination was statistically significant after implementation (p < 0.01).

Table 5 reports the KAP outcomes in the pre- and post-implementation groups. In the pre-implementation phase, eight (33.3%) were good, four (16.7%) were fair, and 12 (50%) were poor. In the post-implementation phase, 20 (83.3%) were good, two (8.3%) were fair, and two (8.3%) were poor. The overall difference in the KAP assessment was statistically significant in the post-implementation phase (p < 0.01).

**Table 5:** KAP outcomes in pre- and post-implementation groups. *: P < 0.05 was considered statistically significant using the Wilcoxon signed-rank test. KAP = knowledge, attitude, and practice

Variable		Pre-implementation (n = 24)		Post-implementation (n = 24)		P-value
		n	%	n	%	
KAP assessment	Good	8	33.3	20	83.3	<0.01*
	Fair	4	16.7	2	8.3	
	Poor	12	50.0	2	8.3	

## Discussion

In this study, we aimed to evaluate the impact of a nursing education program on reducing HAIs in the NICU of a tertiary care center in Pakistan. The results indicate a significant reduction in the rate of HAIs after the implementation of the nursing education program. The rate of HAIs decreased from 10.8% to 5%, representing a 54% reduction. Additionally, the study showed a positive trend in patient outcomes, although not statistically significant.

According to a similar study by Bano et al., before and after the implementation of the guidelines program for the prevention of HAIs, the post-intervention group had the highest mean score of 18.03 ± 1.98 (p = 0.001) [13].

In this study, the difference in the KAP assessment was statistically significant in the post-implementation phase with p < 0.01. In a similar study by Elfiky et al., only 40% of participants were found to have adequate practice while 67% of individuals were deemed to have a satisfactory level of knowledge [14]. Generally, in any LMIC, the difference in practices in private and public sectors may affect the incidence of HAIs. According to Gulia et al., only a moderate grasp of HAI prevention was reported by 70% of NICU nurses. In NICUs, nurse practices for preventing HAI were effective (60%) [15]. Kakkar et al. examined the pre-intervention KAP of 105 nurses. In the places where they were posted, the prevalence of CAUTIs and IV line-related infections was determined. Although not statistically significant (p = 0.15), there was a decrease in the frequency of IV line-related infections [16].

Salem and colleagues conducted a study on the attitudes of healthcare professionals toward infection control strategies in the NICU at Cairo University Hospital. They concluded that a lack of nursing staff, a lack of opportunity for infection control training, and work overload were the main challenges to infection control at NICU [17]. According to Sadeghi-Moghaddam et al., HAI and mortality rates considerably decreased after hand hygiene compliance increased [18].

According to Balla et al., the QI program reduced CLABSI by 89% from the baseline rate. The BSI decreased from 7.3% to 2.3%. Overall mortality decreased over the intervention period, from 2.9% to 1.7% [19].

In another study by Hussain et al., the central line utilization ratio reduced from 0.30 to 0, which also successfully reduced CLABSI rates from 17.1/1,000 device days to 5.0/1,000 device days (p = 0.006) [20]. Pre-intervention and post-intervention data were compared by Bizzarro et al. CLABSI cases per 1,000 central line days in the NICU reduced from 8.40 to 1.28 [21].

Pai et al. reported the participants’ mean knowledge score increased from 21.44 ± 3.06 on the pre-test to 30.26 ± 2.46 on the post-test. Statistically significant differences between the nurses’ pre-test and post-test knowledge and practice scores showed that the educational intervention was successful in improving their knowledge (p = 0.001) and practice (p = 0.001) [22]. In another study, there were highly statistically significant variations between the mean scores of knowledge before and after nursing implementation. It was evident that the nursing implementation guidelines had a positive effect on nurses’ understanding of VAP prevention in newborns [4].

This study had a few limitations. We could not work on environmental factors associated with HAIs. Environmental factors play a very pivotal role in preventive strategies for HAIs in critical care.

## Conclusions

According to this study, nurses’ practices for protecting neonates in the NICU from HAIs have greatly improved. The training session based on the typical guidelines was an efficient and cost-effective intervention to improve the nurses’ practices and reduction of HAIs. There are other contributing factors to HAIs such as the infrastructure of the NICU, isolation policies, and the nurse-to-patient ratio. This gives insights for future research to minimize HAIs in NICUs and thus minimize morbidity and mortality.
